# Lifetime musical training and cognitive performance in a memory clinic population: A cross-sectional study

**DOI:** 10.1177/1029864920918636

**Published:** 2020-06-05

**Authors:** Daisy Fancourt, Katharina Geschke, Andreas Fellgiebel, Alexandra Wuttke-Linnemann

**Affiliations:** Department of Behavioural Science and Health, Institute of Epidemiology & Health Care, University College London, United Kingdom; Department of Psychiatry and Psychotherapy, University Medical Center Mainz, Germany; Center for Mental Health in Old Age, Landeskrankenhaus, Germany

**Keywords:** Arts, cognition, dementia, music, healthy ageing, resilience

## Abstract

**Background:**

Music training has been found to be beneficial for young and healthy participants but the associations between musical training and the cognitive functioning of elderly participants have not been reported consistently. We examined whether lifetime musical training is associated with neuropsychological performance in a memory clinic population of older patients.

**Methods:**

A total of 478 patients (54.2% female, mean age 73.70 ± 6.22, mean Mini Mental State Examination score 25 ± 3) were included in the cross-sectional analyses. All patients were referred to the memory clinic due to cognitive impairments. During the course of diagnosis, all patients underwent neuropsychological tests using the CERAD neuropsychological assessment battery. Patients provided information on whether they ever learned to play an instrument for at least five years in their life.

**Results:**

Neuropsychological test results differed based on musical training (*p* = .042). Overall, there were no differences in any domains of cognitive functioning, other than that patients with musical training performed worse on word list memory (*p* = .008). However, this relationship varied based on the extent of cognitive impairments. Patients who were cognitively unimpaired (Mini Mental State Examination score 27–30) and had musical training showed better word list learning, whereas patients with cognitive impairments (Mini Mental State Examination score < 27) and musical training performed worse in word list learning (*p* = .042) and word list recall (*p* = .045).

**Discussion:**

Overall, there was little evidence of associations between specific neuropsychological test results and musical training. Only in cognitively unimpaired patients was there evidence that musical training had beneficial associations. In patients with cognitive impairment, there were suggestions of negative associations with verbal memory. Future research should longitudinally investigate the beneficial effects of musical training in people with and without cognitive impairments.

## Introduction

There is a wide literature demonstrating the effects of making and listening to music on practically every cognitive function (Zatorre, 2005). Specifically, musical *training* (repeated instruction in music) has been found to have benefits for cognition in diverse populations, including inducing functional and structural cognitive changes (Kraus & Chandrasekaran, 2010). Amongst children, cross-sectional and longitudinal studies have shown associations between music lessons and both improvements in auditory skill and in wider executive function and visual-spatial, verbal and mathematical performance (Degé et al., 2011; Kraus & Chandrasekaran, 2010; Schlaug et al., 2005). Similarly, amongst adults, professional musicians have been found to have enhanced cognitive function (including function specifically related to auditory perception and more general cognitive function; Aleman et al., 2000; Schön et al., 2004) and greater grey matter both in brain regions closely linked to skills learned from musical training and those outside these regions (including the primary sensorimotor cortex, adjacent superior premotor and anterior superior parietal cortex bilaterally, cerebellum and interior frontal gyrus; Gaser & Schlaug, 2003). Notably, it is not just long-term training that has been associated with cognition. Short (20-day) online music training programmes (focusing on listening activities to improve basic musical skills) have been found to improve executive function in children (Moreno et al., 2011). Programmes of one year of instrumental music training have been linked to increases in fine motor skill and auditory discrimination skills (Schlaug et al., 2005), and 15-month programmes of instrumental music training have been correlated with structural brain changes along with improvements in musically relevant motor and auditory skills (Hyde et al., 2009).

In light of these links between musical training and cognition earlier in life, a key question is whether musical training can confer protective effects on cognition in later life, for older adults generally, as well as those with mild-moderate cognitive impairment or severe cognitive impairment. A number of specific intervention studies have found benefits of short-term musical training in these older populations. For older adults *without cognitive impairment*, a small-scale study found that six months of piano lessons improved executive function in those aged 60–85, with effects getting stronger over the ensuing 3-month follow-up period (Bugos et al., 2007). Similarly, a study of six months of music-based multitask training for adults over the age of 65 found improvements in the “sensitivity to interference” subtest of the frontal assessment battery compared to a non-intervention control group (Hars et al., 2014). This study also found preliminary data to suggest that music-based multitask training led to improvements in Mini Mental State Examination (MMSE) score and a reduction in the number of participants who were categorised on this scale as having impaired cognitive performance. For older adults with *mild-moderate cognitive impairment*, one study found that six weeks of cognitive music training for older adults with mild-moderate cognitive impairment led to improvements in MMSE, verbal fluency and spatial perception (Biasutti & Mangiacotti, 2018). Specifically for people with *severe cognitive impairment*, it has been shown that ten weeks of singing training can enhance orientation, remote episodic memory, attention, executive function, short-term and working memory and general cognition (Särkämö et al., 2014). Notably, short-term and working memory were enhanced more by singing training than music listening, highlighting the importance of the training component.

However, such studies are limited in sample size and follow-up. Furthermore, they focus on a broad definition of music training (acquisition of broad musical skills, instrumental lessons, singing) rather than specifically on musical training as defined by learning to play an instrument across the lifespan. When considering results from longitudinal studies that take account of this life-course involvement, results become more mixed. Some have found that learning to play an instrument is associated with a reduced risk of developing cognitive impairment (Balbag et al., 2014; Verghese et al., 2003). But others have not found such associations (Hughes et al., 2010).

Consequently, our understanding of the effects of lifetime musical training on cognition in older adults generally and its association with the transition from healthy cognition to the manifestation of cognitive impairments, specifically, remains limited. Nevertheless, there are a number of reasons why we might hypothesise an association. The auditory perception of music recruits not just neural circuits specific to the musical stimulus but also those that more widely underlie memory, attention, imagery and semantic and syntactic processing (such as bilateral temporal, frontal and parietal neural circuits; Janata et al., 2002). Music training involves the development of rehearsal strategies (a component of sustained attention), which lead to the maintenance of memories (Franklin et al., 2008). Music is also stress reducing (Pelletier, 2004), which is significant as stress has been linked with faster cognitive decline, such as through weakened prefrontal networks, lower systolic blood pressure reactivity or increased cortisol levels (Arnsten, 2015; Pulopulos et al., 2014; Yano et al., 2016). Music can heighten arousal, which has been shown to lead to improved cognitive performance (Thompson et al., 2001). Music can lead to increases in positive emotions (Juslin, 2013) that can increase brain dopamine levels, which are associated with improved cognitive flexibility (Ashby et al., 1999; Blood & Zatorre, 2001; Salimpoor et al., 2011). Music may also affect cognition through indirect means, such as enhancing social engagement (Tarr et al., 2014), which has been shown to have neuroprotective effects through supporting resilience (McFadden & Basting, 2010). Although all of these mechanisms could also apply to broader musical engagement rather than musical training specifically, there are some aspects of musical training that may relate to specific neurocognitive mechanisms. For example, musical training such as learning to play a musical instrument supports structural and functional neuroplastic changes (Schlaug, 2015; Wan & Schlaug, 2010). Even if the musical training in learning to play an instrument is early in life, there is biological evidence of long-term plasticity as a result of this training (White- Schwoch et al., 2013). Further, this instrumental music training may support the transfer of learning from one neuro-cognitive domain to another, enabling specific musical training to have wider effects on cognition (White et al., 2013).

In this regard, Gooding et al. (2014) discuss the proposition that musical training could be one way of increasing cognitive reserve (the resilience of the brain against cognitive decline) and thus propose that it has the ability to postpone the onset of symptoms of age-related cognitive decline. Most interestingly, cognitive functions related to music making seem to appear particularly robust against loss in patients with dementia (Baird & Samson, 2009). Whereas typically cognitive domains such as memory, attention and executive function are impaired in these patients, Baird and Samson (2009) found implicit music memory (e.g., knowing how to play an instrument) to remain intact. Given the shared neural circuits between musical processing and other cognitive and emotional circuits in the brain as described above, it might be plausible to assume that apart from postponing cognitive decline in healthy older adults, there might be beneficial effects of musical training—specifically of instrumental music training—for people with cognitive impairments that transfer to these other domains as well. Therefore, it might be hypothesised that musical training has particular protective effects in patients with cognitive impairments. In other words, if we consider the extreme case of someone who has no cognitive impairment, this person might perform well in neuropsychological tests and the incremental benefit of playing an instrument might be smaller. However, someone who has cognitive impairment might benefit more from musical training due to its assumed shielding effect.

Therefore, this study set out to explore whether specific neuropsychological profiles in older adults differ based on musical training—defined as having learnt to play an instrument—and how these associations vary by the extent of cognitive impairment. Specifically, we hypothesised that (a) neuropsychological profiles would differ based on musical training, with stronger cognitive scores for those with five or more years of lifetime training; and (b) the association between musical training and neuropsychological test profiles would vary depending on the extent of cognitive impairment, with evidence of a greater protective effect of musical training on cognition amongst those with greater levels of cognitive impairment.

## Material and Methods

### Sample characteristics

Anonymous routine data from the outpatient memory clinic of the University Medical Center Mainz, Department of Psychiatry and Psychotherapy, were analysed. Between July 2013 and December 2017, a total of 621 patients underwent comprehensive neuropsychological testing by means of the CERAD Neuropsychological Assessment Battery. Criteria for eligibility in our analyses were: complete results in CERAD subtests (*n*=91 patients provided incomplete test results, of whom *n*=62 completed less than 50% of tests so were excluded) and ⩾ 60 years of age (*n*=52 < 60), resulting in a total sample of 478 patients (54.2% female) with a mean age of 73.70 ± 6.22 (range 60 to 90) (Table 1).

**Table 1.** Sociodemographic and clinical characteristics of study sample.

	Mean ± SD	%
Sociodemographic variables		
Sex (female)		54.18
Age (in years)	73.70 ± 6.22	
Education (in years)	12.33 ± 2.99	
Premorbid intelligence quotient	96.94 ± 11.16	
Musical training for at least five years		21.13
Neuropsychological test results (*z-*scores according to CERAD norm controlling for age, sex and years of education)		
Semantic word fluency	−1.17 ± 1.10	
Boston Naming Test	−0.71 ± 1.45	
Mini Mental State Examination (MMSE)	−2.32 ± 1.68	
Word list learning total	−1.64 ± 1.47	
Word list recall	−1.74 ± 1.34	
Word list recognition	−1.25 ± 1.58	
Visuo-constructional drawing	−0.83 ± 1.35	
Visuo-constructional drawing recall	−1.91 ± 1.29	
Phonematic word fluency	−0.56 ± 1.10	
Depressive symptoms (as measured by GDS-30)		
No depressive symptoms		67.36
Mild depressive symptoms		26.78
Severe depressive symptoms		5.86

SD: standard deviation; GDS-30: Geriatric Depression Scale.

The mean Mini Mental State Examination (MMSE) score was 25 ± 3 (range 12–30) with 37.4% of patients scoring between 27 and 30 (no cognitive impairment), 56.5% of patients scoring between 20 and 26 (mild cognitive impairment), 6.1% of patients scoring between 10 and 19 (moderate impairment) and no patient scoring in the range of severe impairment (MMSE score < 10) according to the guidelines of the German Association for Psychiatry, Psychotherapy and Psychosomatics (DGPPN). The study received ethical approval from the state medical association in Rhineland Palatine (Landesärztekammer Rheinland-Pfalz).

### Material and procedure

All patients completed the German version of the CERAD Neuropsychological Assessment Battery (Chandler et al., 2005). This test battery comprises five subtests: semantic verbal fluency (animals), Boston Naming Test (15 items), MMSE, 10-word list task (learning, recall and recognition), visuo-constructional praxis (drawing, recall) as well as two additional tests: Trail Making Test Parts A and B (TMT A and TMT B) and phonematic verbal fluency (initial letter S).

Musical training was noted using a self-report measure of whether participants had less than five years of musical training versus more than five years of training. Of the study sample, 21.1% (*n* =101) reported having played an instrument for at least five years in their lifetime. In this study, learning to play an instrument was considered musical training whereas singing was not.

Given research suggesting that those who persevere with music training are typically from higher-income, more stable families, have had more education and can have personality traits associated with higher persistence and intelligence (Costa-Giomi, 2012), we included indicators of premorbid intelligence quotient as covariates. Premorbid intelligence was estimated using the formula proposed by Jahn et al. (2013), based on age, sex, birth order (first born/only child: yes/no) graduation, school grades (grade point average, grade point average in maths), highest job position, private internet use (yes/no), newspaper reading (tabloids vs regional press vs national press), book reading (no books vs popular fiction vs nonfiction/textbook vs lyric poetry/essays/classics/scientific reading), current place of residence (< 20,000 inhabitants vs > 20,000 inhabitants) and musical training (never or less than five years of musical training vs more than five years of musical training). The sum score provided by this estimate can be used to predict scores for the German version of the Wechsler Adult Intelligence Scale (Tewes, 1994) with highest confidence for estimating the premorbid verbal IQ and the premorbid full scale IQ (Jahn et al., 2013). When controlling for indicators of premorbid intelligence in our statistical models, we excluded musical training from this list, so we could include it in our statistical models in its own right.

Additionally, given research showing associations between music and depression (Maratos et al., 2008) and further research showing associations between depression and cognition (Wang & Blazer, 2015), we included depressive symptoms as a further covariate. These were assessed by means of the Geriatric Depression Scale (GDS-30) distinguishing no depression (sum score 0–9), from mild depression (sum score 10–19) and severe depression (sum score 20–30). The mean GDS-30 sum score in this sample was 8.24 ± 5.85 (range: 0 to 29).

### Statistical analyses

Data were analysed in a combination of SPSS and R using a multivariate analysis of covariance. First an unadjusted model using *z*-scores as dependent variables was performed (Model 1). In subsequent models we controlled for age, sex and years of education (Model 2), indicators of premorbid intelligence (Model 3) and self-reported depressive symptoms (Model 4) as co-variates. Post hoc analyses were performed to further interpret data.

The raw scores from all neuropsychological test results were transformed into *z*-scores according to the CERAD norm, which controls for age, sex and years of education. We looked at the moderating role of cognitive impairment by adding MMSE bands as a predictor within the model using the interaction term MMSE bands*musical training. In a first step, we used the categorisation of MMSE sum scores as recommended by the guidelines of the DGPPN. As 56.5% of patients had an MMSE score between 20–26 (mild cognitive impairment), we re-ran this model using the MMSE categories as recommended by Folstein et al. (1975) as sensitivity analyses. This classification distinguishes no cognitive impairment (27–30) (37.4%), slight impairment (23–26) (42.9%), moderate impairment (18–22) (17.4%) and severe impairment (<18) (2.3%) and thus leads to a more fine-grained distribution of patients scoring mild-moderate cognitive impairments. To further test the robustness of our moderation results, we performed sensitivity analyses that included MMSE score as continuous rather than categorical variable.

As 9.0% of patients were not able to complete TMT A and 39.3% of patients were not able to complete TMT B in time, TMT A, TMT B and its ratio TMT A/TMT B were excluded from all analyses. Univariate statistics show that in the subsample who completed the TMT, there were no differences in performance based on musical training (all *p* ⩾ .295).

## Results

*Hypothesis 1: Neuropsychological profiles differ based on musical training*

**Figure 1.**
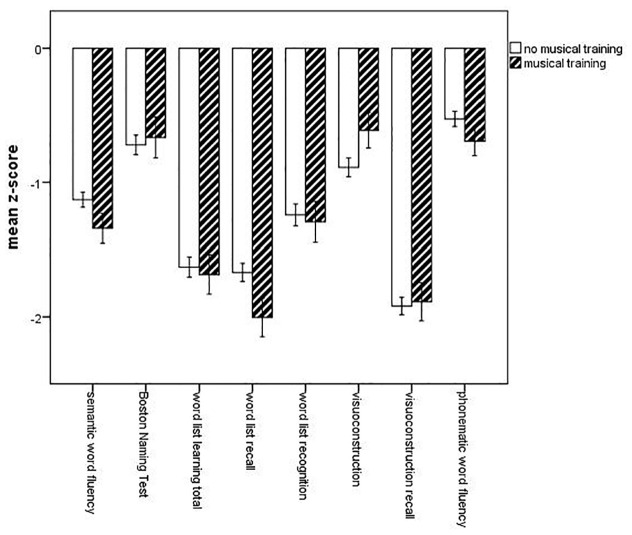
Mean *z*-scores for neuropsychological test results based on musical training. *Z*-scores are standardised using normative data controlling for age, sex and years of education, error bars represent the standard error of the mean. Using multivariate tests, only the performance in word list recall differed based on musical training (*p* = .008).

As hypothesised, in Model 1 (unadjusted model) and 2 (controlling for age, sex and years of education), neuropsychological profiles differed based on musical training (Model 1: *F*[8, 469] = 2.541, *p* = .010, *η²* = .042; Model 2: *F*[8,466] = 2.025, *p* = .042, *η²* = .034; Figure 1).

However, the direction of association was contrary to our hypothesis. Post hoc univariate statistics clarified that performance in word list recall was better for patients without musical training (Model 1: *F*[1,476] = 5.034, *p* = .025, *η²* = .010); Model 2: *F*[1,473] = 7.004, *p* = .008, *η²* = .015). When additionally controlling for premorbid intelligence (Model 3), multivariate tests showed no difference in neuropsychological test profiles based on musical training (*F*[8, 457] = 1.838, *p* = .068). However, univariate statistics showed that the significant effect noted in Models 1 and 2 was maintained (*F*[1, 464] = 6.723, *p* =.010, *η²* = .014). When additionally controlling for depressive symptoms (Model 4), multivariate tests showed no difference in neuropsychological profiles based on musical training (*F*[8, 456] = 1.691, *p* = .098), whereas again univariate tests showed that the advantage in word list recall for patients without musical training was maintained (*F*[1, 463] = 6.012, *p* = .015, *η²* = .013).

Full results for Models 1 to 4 can be found in Supplemental Material (Appendix A).

*Hypothesis 2: the associations between musical training and neuropsychological test profiles vary depending on the extent of cognitive impairment with evidence of a greater protective effect of musical training on cognition amongst those with greater levels of cognitive impairment.*

In all multivariate models, the interaction term MMSE bands*musical training was not significant (all *p* ⩾ .417). However, in univariate tests using the unadjusted model (Model 1), the interaction term considering word list learning total was significant (*F*[2,472] = 3.105, *p* = .046, *η²* = .013). This was maintained in Model 2 (controlling for age, sex and years of education), where post hoc univariate tests showed there was a significant interaction effect of MMSE bands*musical training for word list learning total (*F*[2, 469] = 3.204, *p* = .042, *η²* =.013) and word list recall (*F*[2,469] = 3.115, *p* = .045, *η²* = .013), but it was attenuated in Models 3 and 4.

When using the categorical classification of MMSE as recommended by Folstein (1975), which leads to a more fine-grained distribution of patients scoring mild-moderate cognitive impairments for multivariate analyses, the interaction term MMSE bands*musical training was not significant for any of the models (*p* ⩾ .498). However, post hoc univariate statistics confirmed the significant interaction concerning word list learning total across all models (Model 1: *F*[3,470] = 2.701, *p* = .045, *η²* = .017), Model 2: *F*[3,467] = 2.745, *p* = .043, η*²* = .017, Model 3: *F*[4,458] = 3.886, *p* = .055, *η²* =.016, Model 4: *F*[3,457] = 2.692, *p* = .046, *η²* = .017), whereas the interaction with word list recall did not remain significant in any model (*p* ⩾ .084).

**Figure 2.**
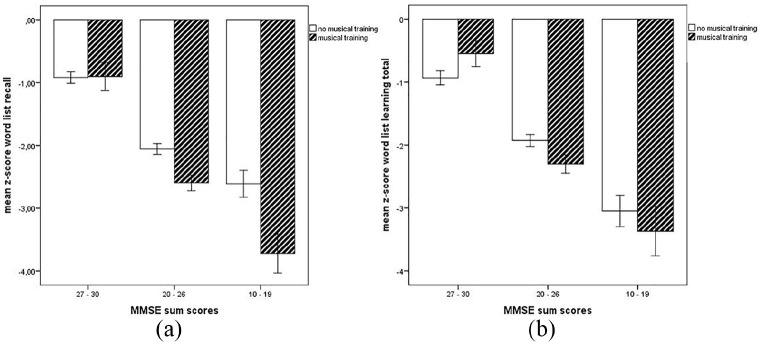
Illustration of significant interactions Mini Mental State Examination (MMSE)*musical training for neuropsychological test results on word list recall (a) and word list learning total (b). No cognitive impairment: MMSE score (categorised according to the bands proposed by DGPPN) 27–30; mild moderate impairment: MMSE score 20–26, moderate cognitive impairment: MMSE score: 10–19, no patients with severe cognitive impairment (MMSE score < 10); error bars represent the standard error of the mean.

Illustration of these interactions using the original DGPPN MMSE bands show that amongst participants with no cognitive impairment there was no difference in word list recall based on musical training (Figure 2[a]), although there was a beneficial association between musical training and word list learning total (Figure 2[b]). For patients with cognitive impairments, musical training was associated with poorer performance on word list learning. When using the MMSE bands as proposed by Folstein (1975), this pattern was confirmed such that patients with no cognitive impairments performed better when having musical training, whereas those patients with musical training and mild to moderate cognitive impairments performed worse.

Sensitivity analyses using MMSE score as a continuous instead of categorical score showed the interaction MMSE*musical training was not significant in any model (*p* ⩾ .639).

However, univariate statistics confirmed a significant interaction for word list recall in model 1 (*p* = .047). The results concerning these sensitivity analyses can be found in the supplemental material (Appendix B).

## Discussion

This study explored associations between lifetime musical training – defined as having learnt to play an instrument for five years or more – and neuropsychological profiles in adults aged 60 and above. We hypothesised that (a) neuropsychological profiles would differ based on musical training, with stronger cognitive scores for those with five or more years of lifetime training; and (b) the association between musical training and neuropsychological test profiles would vary depending on the extent of cognitive impairment with evidence of a greater protective effect of musical training on cognition amongst those with greater levels of cognitive impairment. For Hypothesis 1, we found no associations between musical training and neuropsychological responses, with the exception that participants without musical training performed better on word list recall. Further, our results contradicted Hypothesis 2, showing a protective association only for individuals with no cognitive impairment, but a detrimental association for individuals with cognitive impairment.

Our main finding was that there was little evidence of an association between musical training and cognition amongst our population of memory clinic patients. This null finding goes against some previous positive studies but is echoed by previous research (Hughes et al., 2010). The only finding we did have was that there were mild associations between musical training and performance in word list memory. When considering the extent of cognitive impairment, we found that musical training was associated with better word list learning in healthy participants. But in patients with mild to moderate dementia, musical training was associated with poorer word list learning. Interestingly, most of these results persisted even when controlling for powerful predictors such as years of education and other aspects of premorbid intelligence, suggesting that musical training forms an incremental source of variance in cognitive functioning in old age. This pattern of results found in our analyses contradicts our hypothesis of the buffering effects of musical training on cognition at first sight; however, these findings might be reconciled. Using a resilience framework, our findings might suggest that buffering effects of musical training on cognition are found in healthy participants only. Given the theories of disuse syndrome and cognitive reserve in ageing (Hultsch et al., 1999; Stern, 2012), our findings may be interpreted in a sense that musical training across the lifetime provides cognitive stimulation and increases cognitive reserve and thus postpones cognitive ageing. However, our study only found positive associations between musical training and word list learning in cognitively unimpaired patients. When the critical threshold distinguishing healthy individuals from individuals with cognitive impairment was overstepped, the beneficial effect of musical training was reversed with negative associations between musical training and specific cognitive domains. As we only have cross-sectional data, we can only speculate on longitudinal associations, but it is possible that musically trained individuals might be able to compensate for cognitive decline for a longer period of time. However, when they can no longer compensate for the decline, disease progression is faster. This is a pattern of results that was also described by Wilson et al. (2002), who found that cognitive activity slowed cognitive decline before the onset of dementia but at the same time it was associated with faster decline after the onset of dementia. Thus, our results do not necessarily point to the negative effects of musical training on cognition in patients with cognitive impairment, but suggest the potential importance of a more nuanced interpretation of research on music and cognitive impairment.

To explore this finding further, future research needs to assess the associations between cognitive functioning and musical training longitudinally to understand whether different mechanisms and temporal dynamics underlie ageing depending on musical training. It will also be important to identify which factors predict the course of cognitive decline in musically trained individuals in comparison to individuals without musical training. In particular, shedding light on the temporal dynamics of cognitive ageing depending on musical training is necessary to understand why individuals with musical training performed worse when they had cognitive impairments in comparison to individuals without musical training.

As stated above, the associations between musical training and cognition were mild overall, with associations with specific domains of cognitive functioning. There were some associations between musical training and verbal memory but there were no differences in other aspects of cognitive functioning. However, this might be due to the nature of the CERAD test battery, which does not extensively assess executive functioning and attention in addition to memory functioning. Previous studies have suggested that executive function mediates the relationship between musical training and intelligence (Degé et al., 2011), with musical training specifically shown to enhance executive function in older adults (Bugos et al., 2007). Similarly, musical training has been shown to enhance aspects of attention including selective auditory attention (Strait et al., 2015), again with this shown to be enhanced in older adults with musical training (White-Schwoch et al., 2013). Therefore, although this study found only weak associations between musical training and one specific aspect cognition as captured by CERAD, which is largely focused on memory, whether musical training is positively associated with other aspects of cognition in older age remains to be explored further.

This study has a number of strengths including its large sample size and use of in-depth neuropsychological testing. However, it also has several limitations. The design was cross-sectional in nature and relied on participant self-report of musical training. It is possible that participants may have misremembered their past musical experiences, or (especially in participants with higher levels of cognitive impairment) forgotten previous training. The assessment of musical training was also just a binary variable. As such, it remains unknown whether more years of musical training and/or more regular and daily time spent performing confer greater protective effects on cognition. It also remains unknown at what point in their lifetime participants had musical training and therefore whether more recent musical training confers more benefits than musical training in childhood. In future studies a more thorough investigation of the exact nature of musical training with regard to quality and quantity is necessary. Furthermore, in future studies, the extent of broader musical activities in general (e.g., listening to music or participation in community choirs) should be taken into consideration as well, especially given that research has shown that these activities are associated with various aspects of health independent of musical training (Fancourt et al., 2014; Finn & Fancourt, 2018; Thoma et al., 2011). Further, as our analyses are based on observational rather than experimental data, we cannot assume causality. In our analyses, we used a measure of previous lifetime musical training that had clear temporal precedence and we controlled for the most salient confounding factors. However, it is also possible that people with musical training also had a life history of engagement with other cognitively beneficial activities (such as reading the newspaper or engaging in more social activities; Scarmeas et al., 2001). Finally, this sample drawn from a memory clinic population was already selective in that it treated patients experiencing signs of cognitive impairment. To shed light on differential effects of musical training based on cognitive impairment, future studies should examine patients with cognitive impairment in comparison to healthy control groups. As only the minority of patients reported musical training in this particular sample, future studies should investigate samples from the general population. So, based on the promising results presented here, further (experimental) studies are required to confirm our hypotheses.

In conclusion, cross-sectionally, musical training appears to have some protective associations with verbal memory in cognitively unimpaired patients. However, this study found no evidence of any beneficial associations between musical training and other domains of cognition or amongst individuals with cognitive impairment. Future studies are needed to understand whether other aspects of musical engagement may have more protective associations with cognition.

## Supplemental Material

110918_-_Appendix_A – Supplemental material for Lifetime Musical Training and Cognitive Performance in a Memory Clinic Population: A Cross-Sectional Study Click here for additional data file. Supplemental material, 110918_-_Appendix_A for Lifetime Musical Training and Cognitive Performance in a Memory Clinic Population: A Cross-Sectional Study by Daisy Fancourt, Katharina Geschke, Andreas Fellgiebel and Alexandra Wuttke-Linnemann in Musicae Scientiae

110918_-_Appendix_B – Supplemental material for Lifetime Musical Training and Cognitive Performance in a Memory Clinic Population: A Cross-Sectional Study Click here for additional data file. Supplemental material, 110918_-_Appendix_B for Lifetime Musical Training and Cognitive Performance in a Memory Clinic Population: A Cross-Sectional Study by Daisy Fancourt, Katharina Geschke, Andreas Fellgiebel and Alexandra Wuttke-Linnemann in Musicae Scientiae

## References

[bibr1-1029864920918636] Aleman A. Nieuwenstein M. R. Böcker K. B. E. de Haan E. H. F. (2000). Music training and mental imagery ability. Neuropsychologia, 38(12), 1664–1668. 11074089 10.1016/s0028-3932(00)00079-8

[bibr2-1029864920918636] Arnsten A. F. T. (2015). Stress weakens prefrontal networks: Molecular insults to higher cognition. Nature Neuroscience, 18(10), 1376–1385. 26404712 10.1038/nn.4087 PMC4816215

[bibr3-1029864920918636] Ashby F. G. Isen A. M. Turken A. U. (1999). A neuropsychological theory of positive affect and its influence on cognition. Psychological Review, 106(3), 529–550. 10467897 10.1037/0033-295x.106.3.529

[bibr4-1029864920918636] Baird A. Samson S. (2009). Memory for music in Alzheimer’s disease: Unforgettable? Neuropsychology Review, 19(1), 85–101. 19214750 10.1007/s11065-009-9085-2

[bibr5-1029864920918636] Balbag M. A. Pedersen N. L. Gatz M. (2014). Playing a Musical Instrument as a Protective Factor against Dementia and Cognitive Impairment: A Population-Based Twin Study. International Journal of Alzheimer’s Disease; 836748. 10.1155/2014/836748 PMC4269311 25544932

[bibr6-1029864920918636] Biasutti M. Mangiacotti A. (2018). Assessing a cognitive music training for older participants: A randomised controlled trial. International Journal of Geriatric Psychiatry, 33(2), 271–278. 28401595 10.1002/gps.4721

[bibr7-1029864920918636] Blood A. J. Zatorre R. J. (2001). Intensely pleasurable responses to music correlate with activity in brain regions implicated in reward and emotion. Proceedings of the National Academy of Sciences, 98(20), 11818–11823. 10.1073/pnas.191355898 PMC58814 11573015

[bibr8-1029864920918636] Bugos J. A. Perlstein W. M. McCrae C. S. Brophy T. S. Bedenbaugh P. H. (2007). Individualized piano instruction enhances executive functioning and working memory in older adults. Aging & Mental Health, 11(4), 464–471. 17612811 10.1080/13607860601086504

[bibr9-1029864920918636] Chandler M. J. Lacritz L. H. Hynan L. S. Barnard H. D. Allen G. Deschner M. Weiner M. F. Cullum C. M. (2005). A total score for the CERAD neuropsychological battery. Neurology, 65(1), 102–106. 16009893 10.1212/01.wnl.0000167607.63000.38

[bibr10-1029864920918636] Costa-Giomi E. (2012). Music instruction and children’s intellectual development: The educational context of music participation. In MacDonald R. Kreutz G. Mitchell L. (Eds.), Music, Health and Wellbeing (pp. 339–356). Oxford University Press. https://books.google.co.uk/books?hl=en&lr=&id=vOAUDAAAQBAJ&oi=fnd&pg=PA339&dq=costagiomi+2012+music+health+wellbeing&ots=vvOAtpvvW9&sig=tLnJBWtlvZcaxtpN8PvXC6Ztvfo#v=onepage&q&f=false

[bibr11-1029864920918636] Degé F. Kubicek C. Schwarzer G. (2011). Music lessons and intelligence: A relation mediated by executive functions. Music Perception: An Interdisciplinary Journal, 29(2), 195–201.

[bibr12-1029864920918636] Fancourt D. Ockelford A. Belai A . (2014). The psychoneuroimmunological effects of music: A systematic review and a new model. Brain, Behavior, and Immunity, 36, 15–26. 10.1016/j.bbi.2013.10.014 24157429

[bibr13-1029864920918636] Finn S. Fancourt D . (2018). The biological impact of listening to music in clinical and nonclinical settings: A systematic review. Progress in Brain Research, 237, 173–200. 29779734 10.1016/bs.pbr.2018.03.007

[bibr14-1029864920918636] Folstein M. F. Folstein S. E. McHugh P. R. (1975). ‘Mini-mental state’. A practical method for grading the cognitive state of patients for the clinician. Journal of Psychiatric Research, 12(3), 189–198. 1202204 10.1016/0022-3956(75)90026-6

[bibr15-1029864920918636] Franklin M. S. Sledge Moore K. Yip C.-Y. Jonides J. Rattray K. Moher J. (2008). The effects of musical training on verbal memory, The effects of musical training on verbal memory. Psychology of Music, 36(3), 353– 365.

[bibr16-1029864920918636] Gaser C. Schlaug G. (2003). Brain structures differ between musicians and non- musicians. The Journal of Neuroscience, 23(27), 9240–9245. 14534258 10.1523/JNEUROSCI.23-27-09240.2003 PMC6740845

[bibr17-1029864920918636] Gooding L. F. Abner E. L. Jicha G. A. Kryscio R. J. Schmitt F. A. (2014). Musical training and late-life cognition. American Journal of Alzheimer’s Disease & Other Dementias, 29(4), 333–343. 10.1177/1533317513517048 PMC4074275 24375575

[bibr18-1029864920918636] Hars M. Herrmann F. R. Gold G. Rizzoli R. Trombetti A. (2014). Effect of music-based multitask training on cognition and mood in older adults. Age and Ageing, 43(2), 196–200. 24212920 10.1093/ageing/aft163

[bibr19-1029864920918636] Hughes T. Chang C.-C. H. Bilt J. V. Ganguli M. (2010). Engagement in reading and hobbies and risk of incident dementia: The MoVIES Project. American Journal of Alzheimer’s Disease and Other Dementias, 25(5), 432–438. 10.1177/1533317510368399 PMC2911991 20660517

[bibr20-1029864920918636] Hultsch D. F. Hertzog C. Small B. J. Dixon R. A. (1999). Use it or lose it: Engaged lifestyle as a buffer of cognitive decline in aging? Psychology and Aging, 14(2), 245– 263. 10403712 10.1037//0882-7974.14.2.245

[bibr21-1029864920918636] Hyde K. L. Lerch J. Norton A. Forgeard M. Winner E. Evans A. C. Schlaug G. (2009). Musical training shapes structural brain development. Journal of Neuroscience, 29(10), 3019–3025. 19279238 10.1523/JNEUROSCI.5118-08.2009 PMC2996392

[bibr22-1029864920918636] Jahn T. Beitlich D. Hepp S. Knecht R. Köhler K. Ortner C. Sperger E. Kerkhoff G. (2013). Drei Sozialformeln zur Schätzung der (prämorbiden) Intelligenzquotienten nach Wechsler. Zeitschrift für Neuropsychologie, 24(1), 7–24.

[bibr23-1029864920918636] Janata P. Tillmann B. Bharucha J. J. (2002). Listening to polyphonic music recruits domain-general attention and working memory circuits. Cognitive, Affective & Behavioral Neuroscience, 2(2), 121–140. 10.3758/cabn.2.2.121 12455680

[bibr24-1029864920918636] Juslin P. N. (2013). From everyday emotions to aesthetic emotions: Towards a unified theory of musical emotions. Physics of Life Reviews, 10(3), 235–266. 23769678 10.1016/j.plrev.2013.05.008

[bibr25-1029864920918636] Kraus N. Chandrasekaran B. (2010). Music training for the development of auditory skills. Nature Reviews Neuroscience, 11(8), 599–605. 20648064 10.1038/nrn2882

[bibr26-1029864920918636] Maratos A. Gold C. Wang X. Crawford M. (2008). Music therapy for depression. In Cochrane Database of Systematic Reviews. John Wiley & Sons, Ltd. 10.1002/14651858.CD004517.pub2 18254052

[bibr27-1029864920918636] McFadden S. H. Basting A. D. (2010). Healthy aging persons and their brains: Promoting resilience through creative engagement. Clinics in Geriatric Medicine, 26(1), 149– 161. 20176299 10.1016/j.cger.2009.11.004

[bibr28-1029864920918636] Moreno S. Bialystok E. Barac R. Schellenberg E. G. Cepeda N. J. Chau T. (2011). Short-term music training enhances verbal intelligence and executive function. Psychological Science, 22(11), 1425–1433. 21969312 10.1177/0956797611416999 PMC3449320

[bibr29-1029864920918636] Pelletier C. L. (2004). The effect of music on decreasing arousal due to stress: A meta-analysis. Journal of Music Therapy, 41(3), 192–214. 15327345 10.1093/jmt/41.3.192

[bibr30-1029864920918636] Pulopulos M. M. Hidalgo V. Almela M. Puig-Perez S. Villada C. Salvador A. (2014). Hair cortisol and cognitive performance in healthy older people. Psychoneuroendocrinology, 44, 100–111. 24767624 10.1016/j.psyneuen.2014.03.002

[bibr31-1029864920918636] Salimpoor V. N. Benovoy M. Larcher K. Dagher A. Zatorre R. J. (2011). Anatomically distinct dopamine release during anticipation and experience of peak emotion to music. Nature Neuroscience, 14(2), 257–262. 21217764 10.1038/nn.2726

[bibr32-1029864920918636] Särkämö T. Tervaniemi M. Laitinen S. Numminen A. Kurki M. Johnson J. K. Rantanen P. (2014). Cognitive, emotional, and social benefits of regular musical activities in early dementia: Randomized controlled study. The Gerontologist, 54(4), 634–650. 24009169 10.1093/geront/gnt100

[bibr33-1029864920918636] Scarmeas N. Levy G. Tang M.-X. Manly J. Stern Y. (2001). Influence of leisure activity on the incidence of Alzheimer’s disease. Neurology, 57(12), 2236–2242. 11756603 10.1212/wnl.57.12.2236 PMC3025284

[bibr34-1029864920918636] Schlaug G. (2015). Musicians and music making as a model for the study of brain plasticity. Progress in Brain Research, 217, 37–55. 25725909 10.1016/bs.pbr.2014.11.020 PMC4430083

[bibr35-1029864920918636] Schlaug G. Norton A. Overy K. Winner E. (2005). Effects of music training on the child’s brain and cognitive development. Annals of the New York Academy of Sciences, 1060(1), 219–230. 16597769 10.1196/annals.1360.015

[bibr36-1029864920918636] Schön D. Magne C. Besson M. (2004). The music of speech: Music training facilitates pitch processing in both music and language. Psychophysiology, 41(3), 341–349. 15102118 10.1111/1469-8986.00172.x

[bibr37-1029864920918636] Stern Y. (2012). Cognitive reserve in ageing and Alzheimer’s disease. The Lancet. Neurology, 11(11), 1006–1012. 23079557 10.1016/S1474-4422(12)70191-6 PMC3507991

[bibr38-1029864920918636] Strait D. L. Slater J. O’Connell S. Kraus N. (2015). Music training relates to the development of neural mechanisms of selective auditory attention. Developmental Cognitive Neuroscience, 12, 94–104. 25660985 10.1016/j.dcn.2015.01.001 PMC6989776

[bibr39-1029864920918636] Tarr B. Launay J. Dunbar R. I. M. (2014). Music and social bonding: “Self-other” merging and neurohormonal mechanisms. Frontiers in Psychology, 5. 10.3389/fpsyg.2014.01096 PMC4179700 25324805

[bibr40-1029864920918636] Tewes U. (1994). Hamburg-Wechsler Intelligenztest für Erwachsene: HAWIE-R; Revision 1991. Huber.

[bibr41-1029864920918636] Thoma M. V. Scholz U. Ehlert U. Nater U. M. Costa A. Villalba E. (2011). The psychoneuroendocrinology of music effects on health. Horizons in Neuroscience Research, 6(6), 189–202.

[bibr42-1029864920918636] Thompson W. F. Schellenberg E. G. Husain G. (2001). Arousal, mood, and the Mozart effect. Psychological Science, 12(3), 248–251. 11437309 10.1111/1467-9280.00345

[bibr43-1029864920918636] Verghese J. Lipton R. B. Katz M. J. Hall C. B. Derby C. A. Kuslansky G. Ambrose A. F. Sliwinski M. Buschke H. (2003). Leisure activities and the risk of dementia in the elderly. The New England Journal of Medicine, 348(25), 2508–2516. 12815136 10.1056/NEJMoa022252

[bibr44-1029864920918636] Wan C. Y. Schlaug G. (2010). Music making as a tool for promoting brain plasticity across the life span. The Neuroscientist, 16(5), 566–577. 20889966 10.1177/1073858410377805 PMC2996135

[bibr45-1029864920918636] Wang S. Blazer D. G. (2015). Depression and cognition in the elderly. Annual Review of Clinical Psychology, 11(1), 331–360. 10.1146/annurev-clinpsy-032814-112828 25581234

[bibr46-1029864920918636] White E. J. Hutka S. A. Williams L. J. Moreno S. (2013). Learning, neural plasticity and sensitive periods: Implications for language acquisition, music training and transfer across the lifespan. Frontiers in Systems Neuroscience, 7. 10.3389/fnsys.2013.00090 PMC3834520 24312022

[bibr47-1029864920918636] White-Schwoch T. Carr K. W. Anderson S. Strait D. L. Kraus N. (2013). Older adults benefit from music training early in life: Biological evidence for long-term training-driven plasticity. Journal of Neuroscience, 33(45), 17667–17674. 24198359 10.1523/JNEUROSCI.2560-13.2013 PMC3818545

[bibr48-1029864920918636] Wilson R. S. Bennett D. A. Bienias J. L. Aggarwal N. T. Leon C. F. M. de, Morris M. C. Schneider J. A. Evans D. A. (2002). Cognitive activity and incident AD in a population-based sample of older persons. Neurology, 59(12), 1910–1914. 12499482 10.1212/01.wnl.0000036905.59156.a1

[bibr49-1029864920918636] Yano Y. Ning H. Reis J. P. Lewis C. E. Launer L. J. Bryan R. N. Yaffe K. Sidney S. Albanese E. Greenland P. Lloyd- Jones D. Liu K. (2016). Blood pressure reactivity to psychological stress in young adults and cognition in midlife: The Coronary Artery Risk Development in Young Adults (CARDIA) Study. Journal of the American Heart Association, 5(1), e002718. 10.1161/JAHA.115.002718 PMC4859392 26764414

[bibr50-1029864920918636] Zatorre R. (2005). Music, the food of neuroscience? Nature, 434(7031), 312–315. 15772648 10.1038/434312a

