# Supplementary material for: Lifetime musical training and cognitive performance in a memory clinic population: A cross-sectional study
Source: Music Sci. 2020 Jun 5;26(1):71–83. doi: 10.1177/1029864920918636 (PMC8847981; doi:10.1177/1029864920918636)
Supplement: 110918_-_Appendix_A – Supplemental material for Lifetime Musical Training and Cognitive Performance in a Memory Clinic Population: A Cross-Sectional Study [file 110918_-_Appendix_A.docx]

|  | **Model 1** | | **Model 2** | | **Model 3** | | **Model 4** | |
| --- | --- | --- | --- | --- | --- | --- | --- | --- |
|  | **F(df)** | **p** | **F(df)** | **p** | **F (df)** | **P** | **F (df)** | **p** |
| **Multivariate Tests** | | | | | | | | |
| Constant Term | 116.292 (8,469) | ≤ .001 | 3.562 (8,466) | .001 | 4.963 (8,457) | ≤.001 | 4.679 (8,456) | ≤ .001 |
| Musical training | 2.541 (8,469) | .010 | 2.025 (8,466) | .042 | 1.838 (8,457) | .068 | 1.691 (8,456) | .098 |
| Age |  |  | 3.119 (8,466) | .002 | 3.975 (8,457) | ≤ .001 | 3.782 (8,456) | ≤ .001 |
| Gender |  |  | 4.883 (8,466) | ≤ .001 | 4.060 (8,457) | ≤ .001 | 4.199 (8,456) | ≤ .001 |
| Years of education |  |  | 4.160 (8,466) | ≤ .001 | 2.476 (8,457) | .012 | 2.476 (8,456) | .012 |
| Birth order |  |  |  |  | .970 (8,457) | .459 | .964 (8,456) | .463 |
| Graduation |  |  |  |  | .852 (8,457) | .557 | .819 (8,456) | .586 |
| GPA |  |  |  |  | 1.161 (8,457) | .321 | 1.162 (8,456) | .321 |
| GPA maths |  |  |  |  | 1.859 (8,457) | .065 | 1.895 (8,456) | .059 |
| Highest job position |  |  |  |  | .717 (8,457) | .676 | .752 (8,456) | .645 |
| Private internet use |  |  |  |  | 4.417 (8,457) | ≤ .001 | 4.150 (8,456) | ≤ .001 |
| Newspaper reading |  |  |  |  | 2.015 (8,457) | .043 | 2.014 (8,456) | .043 |
| Book reading |  |  |  |  | 1.933 (8,457) | .053 | 1.881 (8,456) | .061 |
| Current place of residence |  |  |  |  | 2.796 (8,457) | .005 | 2.653 (8,456) | .007 |
| GDS-30 sum score |  |  |  |  |  |  | 1.753 (8,456) | .084 |
|  |  |  |  |  |  |  |  |  |
| **Tests of between-subject effects (source: musical training)** | | | | | | | | |
| Semantic word fluency | 3.012 (1,476) | .083 | .327 (1,473) | .568 | 1.570 (1,464) | .211 | 1.576 (1,463) | .210 |
| Boston Naming Test | .115 (1,476) | .735 | .319 (1,473) | .572 | .005 (1,464) | .946 | .003 (1,463) | .953 |
| Word list learning total | .113 (1, 476) | .737 | .974 (1,473) | .324 | 1.376 (1,464) | .241 | 1.361 (1,463) | .244 |
| Word list recall | 5.034 (1,476) | .025 | 7.004 (1,473) | .008 | 6.723 (1,464) | .010 | 6.012 (1,463) | .015 |
| Word list recognition | .085 (1,476) | .771 | .648 (1,473) | .421 | .574 (1,464) | .449 | .498 (1,463) | .481 |
| Visuoconstruction | 3.328 (1,476) | .069 | 2.287 (1,473) | .131 | 1.590 (1,464) | .208 | 1.388 (1,463) | .239 |
| Visuoconstruction recall | .047 (1,476) | .828 | .208 (1,473) | .649 | .129 (1,464) | .720 | .169 (1,463) | .681 |
| Phonematic word fluency | 1.842 (1,476) | .175 | .295 (1,473) | .587 | .537 (1,464) | .464 | .633 (1,463) | .427 |

*Annotations: F: Pillai trace is reported, musical training (never or less than five years of musical training vs. more than five years of musical training) entered analyses as fixed factor; all other variables entered analyses as co-variates; covariates are based on premorbid intelligence quotient formula as proposed by Jahn et al. (2013): birth order (first born/only child vs. series child), graduation, school grades (grade point average (GPA), grade point average in math), highest job position, private internet use (yes/no), newspaper reading (tabloids vs. regional press vs. national press), book reading (no books vs. popular fiction vs. nonfiction/textbook vs. lyric poetry/essays/classics/scientific reading), current place of residence (< 20.000 inhabitants vs. > 20.000 inhabitant); GDS-30 sum score: Geriatric Depression Scale*
