# Supplementary material for: Lifetime musical training and cognitive performance in a memory clinic population: A cross-sectional study
Source: Music Sci. 2020 Jun 5;26(1):71–83. doi: 10.1177/1029864920918636 (PMC8847981; doi:10.1177/1029864920918636)
Supplement: 110918_-_Appendix_B – Supplemental material for Lifetime Musical Training and Cognitive Performance in a Memory Clinic Population: A Cross-Sectional Study [file 110918_-_Appendix_B.docx]

|  | **Model 1** | | **Model 2** | | **Model 3** | | **Model 4** | | |
| --- | --- | --- | --- | --- | --- | --- | --- | --- | --- |
|  | **F(df)** | **p** | **F(df)** | **p** | **F (df)** | **p** | **F (df)** | | **p** |
| **Multivariate Tests** | | | | | | | | | |
| Constant Term | 87.821 (8, 440) | ≤ .001 | 6.679 (8,437) | ≤ .001 | 6.359 (8,428) | ≤ .001 | 5.604 (8,427) | | ≤ .001 |
| Musical training | 1.762 (8,440) | .083 | 1.221 (8,437) | .285 | 1.267 (8,428) | .259 | 1.253 (8,427) | | .267 |
| MMSE score | 2.285 (144,3576) | ≤ .001 | 2.384 (144,3552) | ≤ .001 | 2.357 (144,3480) | ≤ .001 | 2.355 (144,3472) | | ≤ .001 |
| Musical training*MMSE sum score | .932 (88, 3576) | .659 | .939 (88,3552) | .639 | .898 (88, 3480) | .739 | .894 (88, 3472) | | .749 |
| Age |  |  | 3.830 (8,437) | ≤ .001 | 4.305 (8,428) | ≤ .001 | 4.081 (8,427) | | ≤ .001 |
| Gender |  |  | 8.049 (8,437) | ≤ .001 | 6.058 (8,428) | ≤ .001 | 6.092 (8,427) | | ≤ .001 |
| Years of education |  |  | 5.030 (8,437) | ≤ .001 | 3.153 (8,428) | .002 | 3.146 (8,427) | | .002 |
| Birth order |  |  |  |  | 1.264 (8,428) | .260 | 1.276 (8,427) | | .254 |
| Graduation |  |  |  |  | .567 (8,428) | .805 | .517 (8,427) | | .844 |
| GPA |  |  |  |  | 1.241 (8,428) | .273 | 1.242 (8,427) | | .273 |
| GPA maths |  |  |  |  | 2.389 (8,428) | .016 | 2.417 (8,427) | | .015 |
| Highest job position |  |  |  |  | .896 (8,428) | .520 | .880 (8,427) | | .533 |
| Private internet use |  |  |  |  | 3.066 (8,428) | .002 | 2.715 (8,427) | | .006 |
| Newspaper reading |  |  |  |  | 1.821 (8,428) | .071 | 1.792 (8,427) | | .077 |
| Book reading |  |  |  |  | 1.607 (8,428) | .121 | 1.491 (8,427) | | .158 |
| Current place of residence |  |  |  |  | 2.985 (8,428) | .003 | 2.938 (8,427) | | .003 |
| GDS-30 sum score |  |  |  |  |  |  | 1.728 (8,427) | | .090 |
|  |  |  |  |  |  |  |  | |  |
| **Tests of between-subject effects (source: musical training*MMSE sum score)** | | | | | | | | | |
| Semantic word fluency | .816 (11,447) | .624 | .892 (11,444) | .548 | .752 (11, 435) | .688 | | .775 (11, 434) | .666 |
| Boston Naming Test | .670 (11,447) | .767 | .728 (11,444) | .711 | .634 (11, 435) | .800 | | .647 (11, 434) | .788 |
| Word list learning total | 1.538 (11,447) | .115 | 1.450 (11,444) | .148 | 1.274 (11, 435) | .237 | | 1.323 (11, 434) | .208 |
| Word list recall | 1.826 (11,447) | .047 | 1.795 (11,444) | .052 | 1.618 (11, 435) | .091 | | 1.532 (11, 434) | .117 |
| Word list recognition | .902 (11,447) | .538 | .935 (11,444) | .506 | .835 (11, 435) | .605 | | .833 (11, 434) | .607 |
| Visuoconstruction | .836 (11,447) | .604 | .841 (11,444) | .599 | .916 (11, 435) | .524 | | 1.012 (11, 434) | .435 |
| Visuoconstruction recall | 1.065 (11,447) | .388 | 1.107 (11,444) | .354 | 1.105 (11, 435) | .355 | | 1.092 (11, 434) | .366 |
| Phonematic word fluency | .839 (11,447) | .601 | .809 (11,444) | .631 | .922 (11, 435) | .519 | | .923 (11, 434) | .518 |

*Annotations: F: Pillai trace is reported, musical training (never or less than five years of musical training vs. more than five years of musical training) and MMSE score (continuous variable) entered analyses as fixed factors; all other variables entered analyses as co-variates; covariates are based on premorbid intelligence quotient formula as proposed by Jahn et al. (2013): birth order (first born/only child vs. series child), graduation, school grades (grade point average (GPA), grade point average in math), highest job position, private internet use (yes/no), newspaper reading (tabloids vs. regional press vs. national press), book reading (no books vs. popular fiction vs. nonfiction/textbook vs. lyric poetry/essays/classics/scientific reading), current place of residence (< 20.000 inhabitants vs. > 20.000 inhabitant); GDS-30 sum score: Geriatric Depression Scale*
